# A Corporate Purpose as an Antecedent to Employee Motivation and Work Engagement

**DOI:** 10.3389/fpsyg.2020.572343

**Published:** 2020-09-18

**Authors:** Lars van Tuin, Wilmar B. Schaufeli, Anja Van den Broeck, Willem van Rhenen

**Affiliations:** ^1^Social, Health and Organizational Psychology, Utrecht University, Utrecht, Netherlands; ^2^Research Unit Work Occupational and Organizational Psychology and Professional Learning, KU Leuven, Leuven, Belgium; ^3^Work and Organization Studies, KU Leuven, Leuven, Belgium; ^4^Optentia, North West University, Vanderbijlpark, South Africa; ^5^Productivity and Engagement, Nyenrode Business Universiteit, Breukelen, Netherlands

**Keywords:** corporate purpose, autonomy, motivation, work engagement, self-determination theory, contribution, meaning

## Abstract

It is generally assumed that a corporate purpose aiming to benefit all stakeholders has a positive effect on employee motivation and engagement, but no empirical studies into these specific effects were found. To examine this assumption, a corporate mission and vision matching the definition of a higher purpose were tested in two subsequent studies. The first study (*N* = 270) was a cross-sectional self-report study. The second study included a longitudinal design (*N* = 56) modeling purpose, motivation, and engagement in a cross-lagged panel model over three time-points. The results associated purpose with motivation and engagement. The subsequent longitudinal analysis confirmed the presumed directionality from purpose to engagement, but not to motivation. Hence, while a corporate purpose can be added to the list of antecedents to work engagement, the relationship with motivation, despite the significant association with purpose in the cross-sectional study, remains more complicated. The present study adds to the knowledge of the beneficial effects of a broader purpose and responds to the current surge of interest in purpose as an instrument for sustainable business.

## Introduction

Purpose-driven organizations can change the world (Barton et al., 2016). The debate on the purpose of organizations seems to develop toward a broader purpose-driven leadership (Harrison et al., 2019). In August 2019, the Business Roundtable, representing the largest United States companies, issued a press announcement redefining purpose to include all stakeholders: employees, customers, suppliers, communities, and shareholders (BRT, 2019). To some, the announcement signaled an end to 50 years sway of shareholder value primacy and profit maximization at all costs (Gelles and Yaffe-Bellany, 2019). Others speak of “shareholder value fatigue” (Harrison et al., 2019, p. 2), indicating that raw stockholder capitalism wanes and makes place for companies displaying true social responsibility by being good and doing well (Husted, 2016).

Popular business publications and consultancy reports frequently applaud the positive effects of a broader purpose on employee motivation and engagement. In the same breath, many of these publications stress that motivation and engagement are in crisis but indispensable for companies to thrive in a Volatile, Uncertain, Complex, and Ambiguous (VUCA) world (cf. Mann and Harter, 2016). One would expect to find studies examining the effects of a corporate purpose on motivation and engagement. However, academic leadership studies often overlook the role of purpose and take it for granted (Kempster et al., 2011). Moreover, empirical research into the psychological effects of a corporate purpose on motivation and engagement is lacking (Parmar et al., 2017). Many references in business (Hurst et al., 2016; Ebert et al., 2018) and academic publications (cf. Shuck and Rose, 2013) point to the beneficial effects of a personal purpose on meaning in life, well-being, and performance. It is also assumed that identification with a broader corporate purpose will foster well-being and engagement. However, they do not provide empirical substantiation for that relationship. Some do present an empirical relationship (cf. Sparks and Schenk, 2001; Steger et al., 2012), but although the authors refer to a higher purpose, they do not measure such a purpose. That is, Sparks and Schenk (2001, p. 860) measure the belief in a higher purpose and define it as “feeling part of a ‘cause’ that is about more than making money.” Moreover, Steger et al. (2012) present a scale measuring positive meaning in work, work as a lever for meaning-making, and the perception of contributing to some greater good through work, without defining what that greater good may be. We consider it essential to complement the debate with empirical knowledge about the actual psychological associations of a real corporate purpose that matches a definition of a broader or higher purpose, with motivation, and engagement.

Based on self-determination theory (Deci and Ryan, 2000), the present study examined the associations of a corporate purpose with employee motivation and work engagement testing the widespread assumption that a higher purpose leads to enhanced employee motivation and engagement. Additionally, we were interested in what role motivation plays in the dynamic between a purpose as an antecedent with engagement as an outcome. We argue that motivation may explain this relationship, in the same manner that basic psychological needs explain the relationship between, e.g., transformational leadership and work engagement (Kovjanic et al., 2012), or between team-values and work engagement (Schreurs et al., 2014).

This study adds to the knowledge of the potential benefits of a corporate purpose and its relations with motivation and engagement. It contributes to the current understanding of the antecedents of employee well-being and answers to the growing interest in more eudemonic forms of well-being (Ilies et al., 2005; Ryan et al., 2008) and sustainable motivation (Peters et al., 2018). Moreover, the study adds to leadership theory and responds to the lack of research into corporate purpose (cf. Podolny et al., 2004). It provides some support for resolving the underutilization of purpose as an instrument to sustainably motivate employees and drive work engagement (Keller, 2015). Finally, considering purpose as an aspect of good leadership (Shuck and Rose, 2013), the study adds to the knowledge about the underlying process that may explain the link between leadership and engagement (Inceoglu et al., 2018). From a practical point of view, this study may provide arguments for organizations to reflect on why they do what they do and how this may affect motivation.

### Defining Purpose

For long, various scholars have advocated the transition from the doctrine of shareholder value (Friedman, 2007) to a broader purpose-driven leadership that considers all stakeholders (Freeman et al., 2004). Recent business studies provide empirical support for the beneficial impact of purpose on business results. Companies pursuing a higher purpose have the better case compared to companies primarily seeking profit maximization (Thakor and Quinn, 2013). Gartenberg et al. (2016) found a broader purpose, combined with clarity and systematic communication around it, predicts financial performance. In addition, Keller (2015) corroborated the business case for a corporate purpose but also concluded that purpose is yet much underutilized.

A broader corporate purpose is generally defined as the meaning and contribution of a firm beyond its financial strategy and performance (Henderson and Steen, 2015). It should involve all stakeholders and put people first (Sisodia and Gelb, 2019), aim to benefit customers (Ellsworth, 2002), integrate the needs of society (Metcalf and Benn, 2012), foster employee well-being and engagement (Bajer, 2016), and include and embrace ethics (Freeman, 1994). Beyond the formal wording of a purpose, it should also be actively propagated: a purpose is only as strong as that employees and other stakeholders believe in it (Bekke, 2006). A compelling purpose instills the organization with value and, through actively supporting employees to identify with and find meaning in it, stimulates commitment and inspires action (Ellsworth, 2002).

Larry Fink, CEO of Blackrock, titled his 2019 letter to CEOs “Purpose and Profit” (Fink, 2019) and claimed that a purpose should unify management, employees, and communities alike and drive ethical behavior. As governments fail to do so, Fink (2019) posits that businesses and organizations are called on to set higher, more exacting standards and allow for the public to hold them accountable. Like Ellsworth (2002), Fink presents purpose as the principal raison d’être of a company, providing a fundamental framework to benefit others and reap sustained long-term rewards. He considers it indispensable for leaders to, through purpose, provide direction and responsible stewardship in times of political polarization and economic disruption.

### Purpose and Motivation

A broader corporate purpose may affect motivation and elicit a sense of meaning and well-being in employees (Gartenberg et al., 2016). Self-determination theory (SDT, Deci and Ryan, 2000) offers a perspective on the underlying mechanism that may explain how environmental aspects, such as a corporate purpose, may lead to higher levels of intrinsic motivation and well-being. At the core of SDT lies the assumption that human beings are active social agents that take in life experiences in social contexts and integrate these with their sense of self, thus making meaning and developing a more unified sense of self-identity (Deci and Ryan, 2000). An appealing corporate purpose serving a broader interest in the pursuit of a greater good may thus support individuals to identify with that purpose and integrate it with their sense of self, which then nourishes high-quality motivation. Moreover, notwithstanding the paucity of studies, motivation and engagement are sought-after qualities in organizations. They are essential to attract and retain talented workers (Delaney and Royal, 2017) and younger generations (Eversole et al., 2012).

This integration process of a corporate purpose with the self is a very important aspect. It cannot be properly understood without an idea about the beliefs people hold about self-identity in life and work. It is a commonly-held belief that a life with a purpose is a life of meaning, happiness, and well-being (Frankl, 2008; Wong, 2012). Work also carries purpose and, over the decades, has increasingly become a principal place for self-expression and self-realization (Ciulla, 2000). Work is a stronghold for the Western ideal of authenticity and self-determination (Taylor, 1991). Work may confirm, strengthen, or deny one’s sense of self and identity (Niemiec and Spence, 2016; Fukuyama, 2018).

Consequently, the expectations people have from work and the work environment are high. The current prevalence of purpose and self-realization makes corporate purpose an essential subject of interest when studying motivation and engagement (Shuck and Rose, 2013). A corporate purpose may support motivation and foster work engagement when tapping into the prevailing beliefs people hold about work and self-realization through work. In a recent study, Martela and Pessi (2018) argue that a broader purpose imbues a sense of autonomy, self-determination, contribution, and worthiness in the individual, to whom the intrinsic value of the work itself is reinforced by the perceived intrinsic value of that broader purpose.

SDT researchers explain the process of integration of purpose with self-identity as the internalization of extrinsic motives (Ryan, 1995; Ryan and Deci, 2017) and distinguish different levels of integration and corresponding types of motivation (Gagné and Deci, 2005). The more an individual can identify with a given purpose, for example, because he or she finds it an essential or inspiring objective to contribute to, the more self-determined the individual may feel. The type of motivation where extrinsic motives, such as a corporate purpose, are effectively internalized with the sense of self is labeled autonomous motivation. This type of motivation positively associates with work engagement (Meyer and Gagné, 2008). Employees displaying high levels of autonomous motivation tend to willingly take on tasks and responsibilities as these align with what they find important (Van den Broeck et al., 2013). When employees find joy in their work and when the work itself is engaging and exciting to them, they can direct their motivational energy for the sake of doing the work itself (Ryan and Deci, 2017). Furthermore, intrinsic motivation is related to enhanced creativity (Amabile, 1985), problem-solving capacities (Song and Grabowski, 2006), self-regulation (Niemiec and Spence, 2016), and taking on responsibility and initiative (Grant et al., 2011). A well-defined corporate purpose can support employees in finding meaning and purpose in the work they are doing and, hence, support their autonomous motivation.

In contrast, the type of motivation where the extrinsic motives are not, or only partially internalized, is defined as controlled motivation. This type of motivation implies that the reason an individual performs a task is less for the sake of the activity itself, but rather to obtain a social or material reward or prevent negative consequences (Gagné et al., 2014). More specifically, when being motivated in a controlled way, employees feel pressured by others or pressure themselves to do their work. Managers who push for deadlines and delivery on KPIs (Key Performance Indicators) or that demand extensive reporting and micro-manage employees are experienced as controlling. However, contingent performance evaluations, strict processes, procedures, or monetary rewards can also be experienced as controlling (Gagné and Deci, 2005). High levels of controlling regulations relative to low autonomous motivation are associated with lower well-being, work-related strain and burnout (Van den Broeck et al., 2013), procrastination (Vansteenkiste et al., 2009), and lower work engagement (Howard et al., 2016).

When individuals describe the reason for performing a task as meaningless or pointless, this is described as amotivation (Deci and Ryan, 2000). Consequently, individuals may experience a lack of control over the situations they are in or feel detached from their work or the actions they undertake (Howard et al., 2016). Known associations of this type of motivation are low engagement, low vitality, emotional exhaustion, higher burnout risk, and turnover intentions (Tremblay et al., 2010).

Hence, it can be expected that purpose positively associates with autonomous motivation, and negatively with amotivation and controlled motivation.

*Hypothesis* 1: *A higher corporate purpose associates positively with (a) autonomous motivation and negatively with (b) controlled motivation, and (c) amotivation.*

### Purpose and Work Engagement

Work engagement is a central concern to organizations. Work shifted from mechanistic to knowledge-intensive models, and social interaction between employees, their well-being, and engagement contribute to the organization’s performance (Shuck and Herd, 2012). The rising importance of work to provide in the search for meaning (Ciulla, 2000) and the competition between organizations to source talented and motivated employees (Delaney and Royal, 2017) add to the complexity. Engaged employees show high levels of energy (Schneider et al., 2018) and self-efficacy (Bandura, 2010). They experience their work as fun and may lose track of time at work (Bakker and Demerouti, 2008). Additionally, they display enhanced levels of well-being (Peters et al., 2018) and report a healthier work-life balance (Kossek et al., 2014).

A robust body of research has developed over the past two decades identifying various antecedents to engagement, such as leadership (Carasco-Saul et al., 2015), work climate (Bakker et al., 2007), and organizational support (Saks, 2006). On an aggregated organizational level, Macey and Schneider (2008) showed that job attributes (variety, challenge, and autonomy) and leadership act as the main antecedents of engagement. The authors position work engagement as a potential key to competitive advantage. Others point to the relationship between business performance and work engagement (e.g., Sorensen, 2013) and even long-term sustainable performance (e.g., Renee Baptiste, 2008).

In a conceptual paper, Shuck and Rose (2013) argue to complement the dominant focus on performance in work engagement studies (Inceoglu et al., 2018) with the development of favorable conditions to nurture engagement through providing meaning and purpose. Following Shuck and Rose’s argument, we hypothesized that purpose would positively associate with work engagement.

*Hypothesis* 2: *Purpose associates positively with work engagement.*

Furthermore, Shuck and Rose’s argument is in keeping with SDT as a unifying framework underlying and potentially explaining work engagement as a phenomenon (Meyer and Gagné, 2008; Vansteenkiste and Ryan, 2013). Following the typology of motivation as described in SDT, and following the argument of Martela and Pessi (2018) that a broader purpose imbues a sense of self-determination in the individual, we expected that autonomous motivation mediates the relationship between purpose and engagement and that amotivation and controlled motivation would not mediate. Previous studies consistently found that needs satisfaction mediates the relationship between the antecedent and outcomes, be it in leadership studies (e.g., Kovjanic et al., 2012), sports (e.g., Gillet et al., 2009), or parenting (e.g., Van der Kaap-Deeder et al., 2017). Nevertheless, studies examining the mediating role of controlled and autonomous motivation are rare. However, Grant et al. (2011), in their study into the moderating role of controlled and autonomous motivation in predicting performance, considered it likely to occur.

*Hypothesis* 3: *(a) Autonomous motivation mediates the relationship between purpose and work engagement, (b) amotivation, and (c) controlled motivation mediate negatively between purpose and engagement.*

### This Study

**Figure 1:**
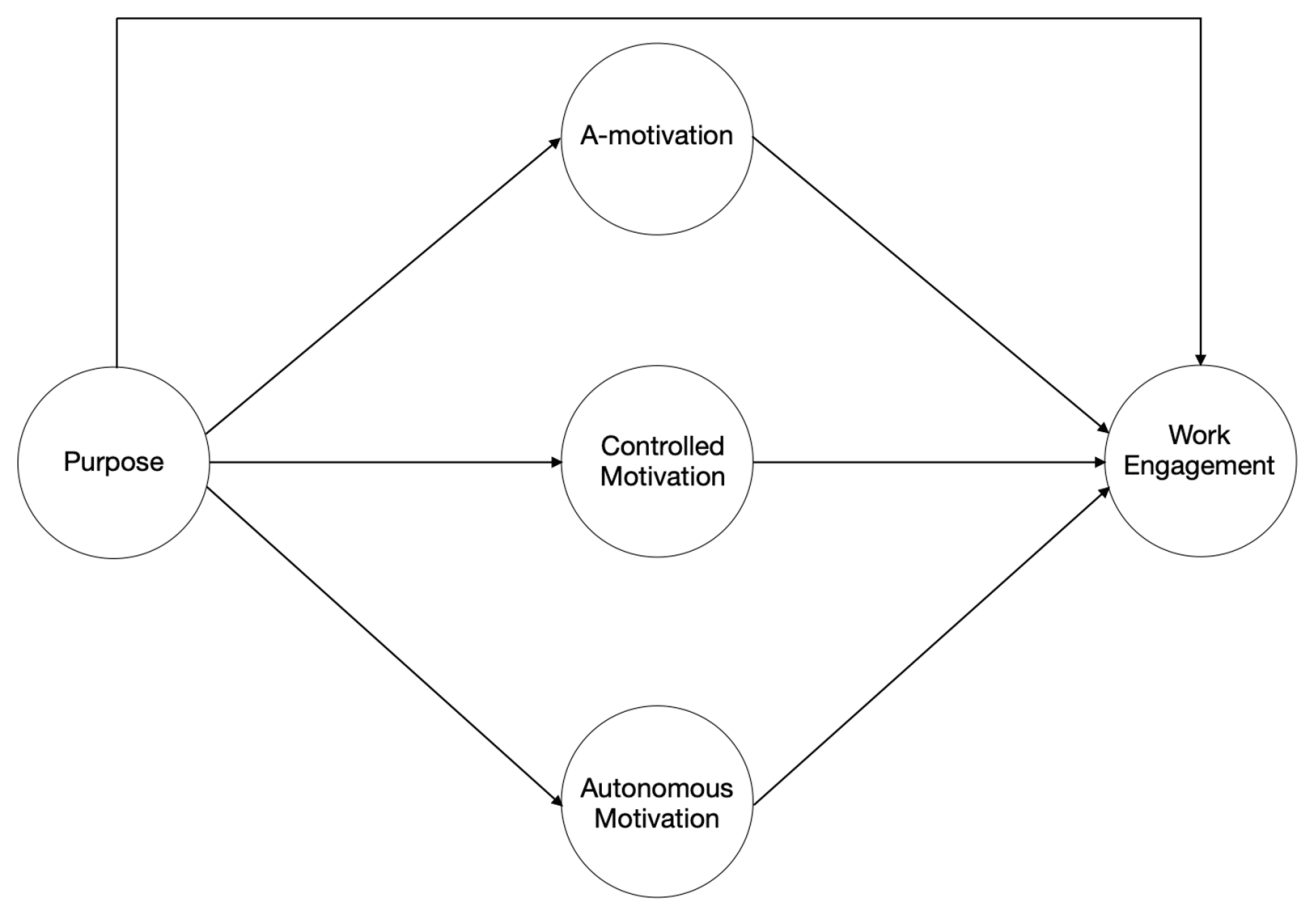
Research model for Study 1. The research model portrays the multiple mediation model of the present study, testing the hypothesis that purpose associates positively with work engagement through autonomous motivation and negatively through controlled motivation and amotivation.

**Figure 2:**
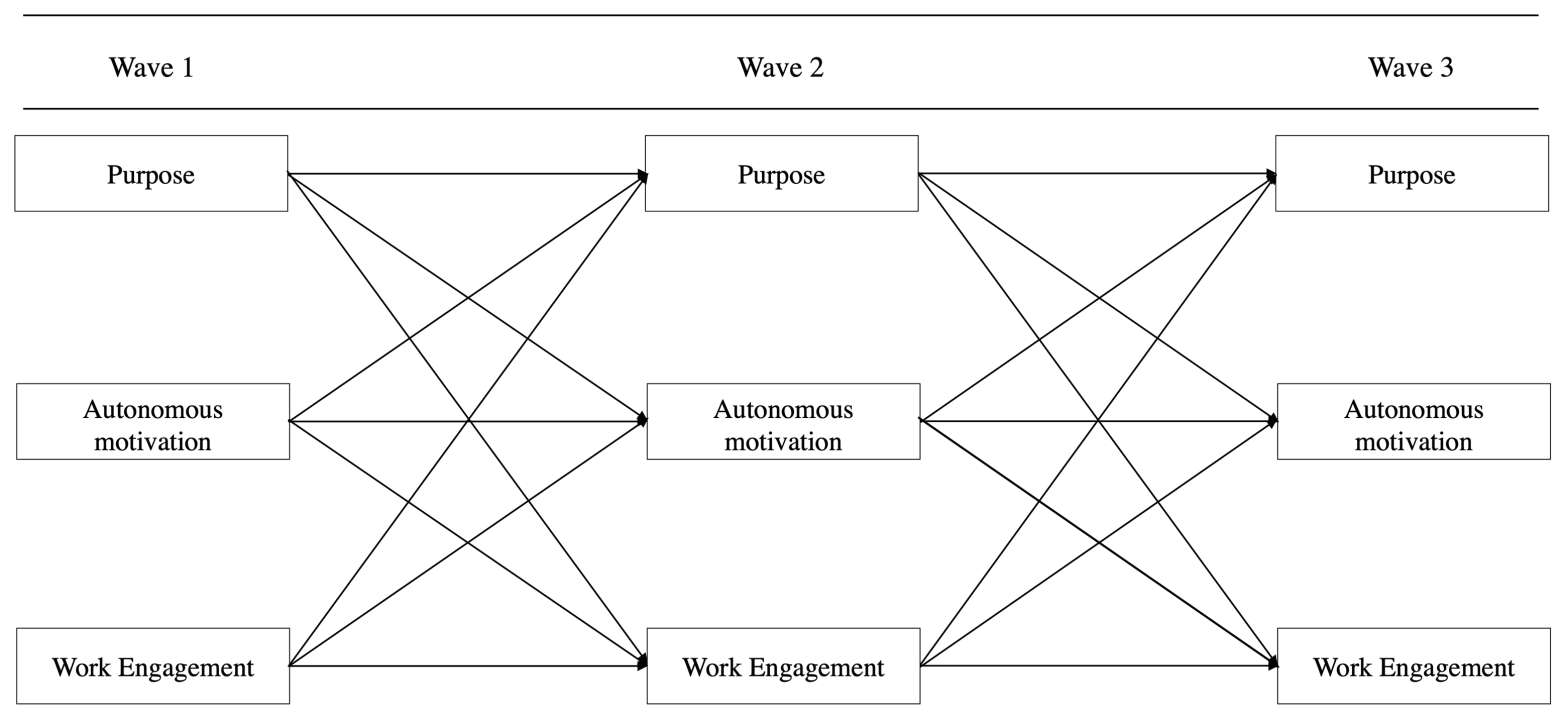
The cross-lagged panel model as used in Study 2 examines the crossed relationships from purpose to autonomous motivation and engagement.

In the present two-step study, we took the corporate purpose of a multinational organization as a point of vantage and tested the associations with employee motivation and work engagement through a cross-sectional self-report survey, following the recommendations for cross-sectional research (Spector, 2019). The selected purpose matched the criteria of a broader or higher purpose and consisted of the organization’s mission and vision statement. The mission statement referred to contributing to improving people’s lives. The vision statement included contributing to health and sustainability, being a great place to work, inspiring passion for the firm’s contribution, and delivering value to customers and shareholders. We asked participants whether the purpose inspired them (Martela and Pessi, 2018) and whether they felt they were contributing to its realization through their work (Steger et al., 2012). The data were specified in a structural model (Figure 1) to simultaneously examine the associations of a corporate purpose with motivation and engagement. Additionally, we examined the potential mediational effects of motivation on the relationship between purpose and engagement. In Study 2, the variables purpose, motivation, and engagement were specified in a cross-lagged panel model (Figure 2) to examine the potential directionality over three time-points with a subset of respondents from the cohort of Study 1.

*Hypothesis* 4: *Purpose relates to autonomous motivation and engagement over time, rather than the other way around.*

## Study 1

### Method

#### Participants and Procedure

Participants were a convenience sample of back-office workers specialized in order management and information analysis from a Dutch for-profit multinational organization producing, selling, and maintaining professional health systems. The supervising managers were informed about the survey and its purpose and agreed on inviting the employees through email. It was also agreed that participation should be voluntary and that there should be no incentives to complete the survey beyond the email invitation itself. Furthermore, the survey was checked for compliance with the survey protocol established by the organization’s works council, guaranteeing the confidentiality and anonymity. Then, the invitations were sent to 432 prospective respondents to complete an online self-report survey. In total, 277 completed responses were received (64%). The average age was 42.23 (*SD* = 10.42), and 43% was female. Of the respondents, 90% had a full-time contract, 9% of which were temporary contracts. The percentage of part-time workers was 10%, of which 8.7% had a temporary contract. Sixty-eight percent of the employees had been in their positions for less than 5 years, 27% between 5–10 years, 4.5% 10 years or longer.

#### Instruments

The measures were estimated on a Likert scale from 1 (*strongly disagree*) to 5 (*strongly agree*) except for work engagement. Reliabilities are reported through Cronbach’s alpha (*α*), congeneric reliability (*ρ* *_C_*), and average variance extracted (AVE, Peterson and Kim, 2013; Cho, 2016; Hair et al., 2017). The items and their factor loadings are presented in Table 1.

**Table 1:** Scale items, factor loadings, and reliabilities.

Scale	Item	*b*	*α*	*ρ_C_*	*AVE*
Purpose	This mission and vision inspires me.	0.58	0.85	0.86	0.56
	I feel that I contribute to (…)^a^ in my daily work.	0.74			
	We strive to make the world healthier.	0.77			
	We strive to make the world more sustainable.	0.70			
	(…)^a^ is the best place to work.	0.63			
	We deliver superior value to our customers.	0.73			
	We deliver superior value for our shareholders.	0.71			
Amotivation	I do not, because I really feel that I’m wasting my time at work.	0.86	0.81	0.89	0.73
	I do not, because I do not think this work is worth putting efforts into.	0.87			
	I do not know why I’m doing this job, its pointless work.	0.83			
Controlled motivation	Because others will reward me financially only if I put enough effort in my job (e.g., employer, supervisor, …).	0.74	0.68	0.76	0.54
	Because others offer me greater job security if I put enough effort in my job (e.g., employer, supervisor…).	0.45			
	Because I risk losing my job if I do not put enough effort in it.	0.92			
Autonomous motivation	Because I have fun doing my job.	0.87	0.85	0.91	0.77
	Because what I do in my work is exciting.	0.88			
	Because the work I do is interesting.	0.87			
Work engagement	At my job, I feel strong and vigorous.	0.86	0.80	0.88	0.72
	I feel happy when I am working intensely.	0.81			
	I am proud of the work that I do.	0.87			

a For reasons of anonymity, the name of the organization and the mission are withheld.

*b*, factor loadings; *α*, Cronbach’s alpha; *ρ_C_*, congeneric reliability; *AVE*, average variance extracted.

*Purpose* was assessed by presenting the organization’s mission and vision to the respondents, followed by seven items that were composed of keywords from the deconstructed mission and vision statements. The items were (1) “I am inspired by this mission and vision”, (2) “I feel that I contribute to (…)^1^ in my daily work,” (3) “We strive to make the world healthier,” (4) “We strive to make the world more sustainable,” (5) “(… see Footnote 1) is the best place to work,” (6) “We deliver superior value to our customers,” and (7) “We deliver superior value for our shareholders.” *α* = 0.85, *ρ* *_C_* = 0.86, *AVE* = 0.56.

For work motivation, we used items from the multidimensional work motivation scale (Gagné et al., 2014) to measure three types of motivation (amotivation, controlled motivation, and autonomous motivation) with three items each. The header for the scale was, “Why do you or would you put efforts in your job?” An example of an item for amotivation is, “I do not, because I really feel that I’m wasting my time at work.” Reliabilities were *α* = 0.81, *ρ* *_C_* = 0.89, *AVE* = 0.73. An example of controlled motivation is “Because others offer me greater job security if I put enough effort in my job,” *α* = 0.68, *ρ* *_C_* = 0.76, *AVE* = 0.54. In addition, an example of autonomous motivation: “Because the work I do is interesting,” *α* = 0.85, *ρ* *_C_* = 0.91, *AVE* = 0.77.

*Work engagement* was assessed using the 9-item version of the Utrecht Work Engagement Scale, UWES (Schaufeli et al., 2006), which measures vigor, dedication, and absorption. Following Schaufeli et al. (2006) recommendations, one common factor for engagement was used (*α* = 0.80, *ρ* *_C_* = 0.88 *AVE* = 0.72) and it was measured on a Likert scale ranging from 0 (*never*) to 6 (*every day*). Examples of items are “At my job I feel strong and vigorous” (vigor); “I am proud of the work that I do” (dedication); and “I feel happy when I am working intensely (absorption).”

### Results

#### Preliminary Analyses

First, the data were checked for missing values, which was <1%. Outlier analysis plotting Cook’s distances and centered leverage resulted in eliminating seven cases from the analysis so that further analyses were conducted with *n* = 270. Then, to test whether data were missing completely at random, Little’s MCAR test (Little and Rubin, 2002) was applied, which showed that MCAR was not violated (*χ*^2^ (51) = 61.50, *p* = 0.15). No effects were found for age, gender, tenure, or type of contract. The means, standard deviations, and bivariate correlations are presented in Table 2. Notably, the Pearson correlations of controlled motivation were all insignificant.

#### Analysis

The structural research model comprised purpose, amotivation, controlled motivation, autonomous motivation, and work engagement, which were tested simultaneously with their respective items. The estimator for the mean- and variance-adjusted likelihood ratio was set to maximum likelihood. To evaluate model fit a range of fit-indices was used following (Marsh and Balla, 1994; Kline, 2016): the chi-square (*χ*^2^); the root mean square error of approximation (RSMEA); and the comparative fit index (Bentler, 1990) in combination with the standardized root mean square residual (SRMR). The model had an acceptable fit to the data: *χ*^2^ (160) = 326.14, *p* < 0.001; *RMSEA* = 0.06, 90% CI [0.05, 0.07]; *CFI* = 0.93; *SRMR* = 0.08 and explained 56.7% of the variance in work engagement.

#### Hypothesis Testing

**Figure 3:**
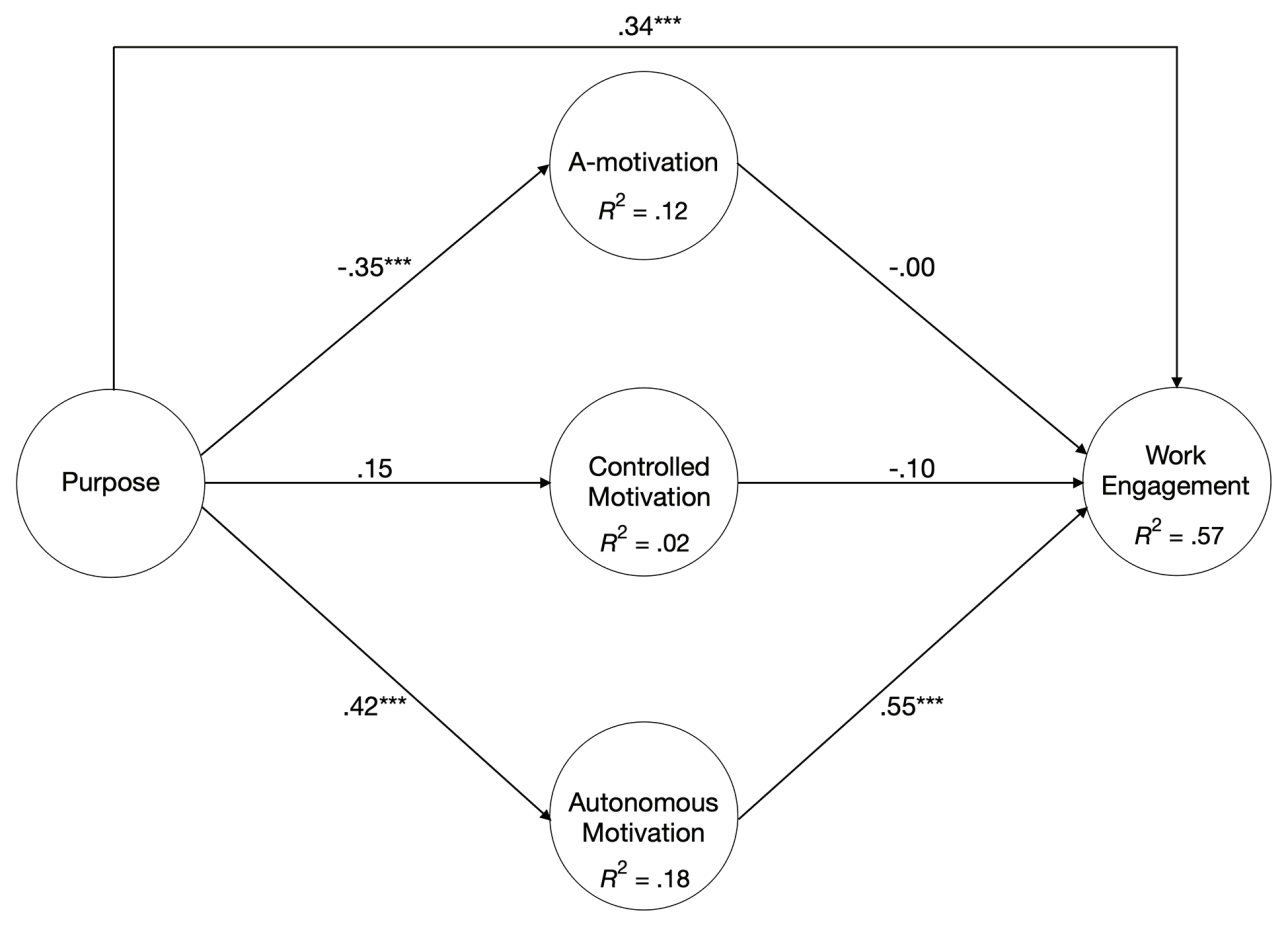
Standardized path coefficients from purpose to work engagement *via* amotivation, controlled motivation, and autonomous motivation.

Hypothesis 1 predicted purpose to associate positively with (a) autonomous motivation and negatively with (b) amotivation, and (c) controlled motivation. The results (see Figure 3) indicate a positive and significant path from purpose to autonomous motivation (β = 0.42, *p* < 0.001), while the path to amotivation is significant and negative (β = −0.35, *p* < 0.001) and the path to controlled motivation is insignificant but positive (β = 0.15). Hence, Hypothesis 1a,b are supported by the data, while Hypothesis 1c is not.

Hypothesis 2 posited that purpose positively associates with work engagement. This is also supported by the data (β = 0.34, *p* < 0.001). Hypothesis 3 stated that (a) autonomous motivation mediates the relationship between purpose and work engagement, which was supported by the data. As depicted in Table 3, the relationship between purpose and work engagement was partially mediated by autonomous motivation (β = 0.23, *p* < 0.001, 95% BCa CI [0.14, 0.33]), whereas there was no mediational role for (b) amotivation, and (c) controlled motivation, which was not expected. To examine the impact of autonomous motivation on the percentage of variance explained in work engagement, another test was carried out specifying only the direct effects of purpose on work engagement. This test resulted in a total variance explained of 18.8% against 56.7% of the proposed and tested model, which underscored the fundamental role of autonomous motivation in the model.

**Table 2:** Means (*M*), standard deviations (*SD*), and bivariate correlations (*r*).

		*M*	*SD*	1	2	3	4	5
1	Purpose	3.61	0.59	1				
2	Amotivation	1.56	0.63	−0.26^***^	1			
3	Controlled motivation	2.84	0.69	0.11	0.08	1		
4	Autonomous motivation	3.77	0.66	0.31^***^	−0.46^***^	−0.05	1	
5	Work engagement	4.74	0.96	0.49^***^	−0.37^***^	−0.03	0.59^***^	1

*n* = 270; significance (two-tailed).

*** *p* < 0.001.

## Study 2

The second study aimed to examine directionality between the study variables purpose, autonomous motivation, and work engagement. Amotivation and controlled motivation were not tested because the first study indicated that these two variables did not mediate.

### Method

Participants were selected from a subset of the participants in Study 1 and contained the order managers of the customer fulfillment center of that same organization. Data were gathered over three waves with an eight-month interval. E-mail invitations were sent to 163 prospective respondents by their supervisors to complete an online self-report survey. Analogous to Study 1 participation was voluntary, and no incentives for participation were issued. At the first wave, 119 completed responses were received (73%) against 120 at wave 2 (74%), and 81 at wave 3 (50%). The subsequent analysis of the data was performed with 56 same respondents. Of the respondents at wave 1, 47% were female, the average age was 39 years (*SD* = 10.31), and the average tenure was 4 years or less. Most employees (67%) had a full-time contract, of which 11% had a temporary arrangement, and 33% worked part-time (32 h per week or less), of which 34% had a temporary contract. There were no effects of gender, age, tenure, or type of contract.

#### Measures

The measures applied were the same as in Study 1: purpose, autonomous motivation, and work engagement. Reliabilities at the subsequent time points were expressed in Cronbach’s alpha, congeneric reliability, and average variance extracted. All values were within acceptable limits. Cronbach’s alpha values varied between 0.76 for work engagement at wave 1 and 0.87 for autonomous motivation at wave 3. Values for congeneric reliability varied between 0.82 for purpose at wave 1 and 0.92 for autonomous motivation at wave 2. Average variance extracted varied between 0.49 for purpose at wave 2 and 0.79 for autonomous motivation at wave 2.

### Results

#### Preliminary Analysis

Missing data analysis indicated that 0.7% of data on the used variables were missing. MCAR was tested with the three variables over the three time points and was not violated (*χ* ^2^(52) = 51.83, *p* = 0.48), and dropout due to systematic attrition was therefore presumed not to have occurred (Asendorpf et al., 2014). Nevertheless, it was examined whether respondents would be more likely to drop out of the study related to one or more of the three variables used in the study. Drop out was defined as all respondents that dropped out at wave 2 or 3. Respondents who completed the survey at wave 1 and 3, but not at wave two, were considered to have stayed on. The effect sizes of the systematic attrition analysis indicated that respondents with lower means for work engagement had a slightly higher chance of dropping out of the study at later waves (*d* = 0.14). Following the recommendations in Asendorpf et al., when MCAR is not violated, it was decided not to correct the data by multiple imputations.

The means, standard deviations, and intercorrelations are depicted in Table 4.

**Table 3:** The relationship between purpose and work engagement as mediated by autonomous motivation.

	*β*	*SD*	95% BCa CI	
			2.50%	97.50%
Total effects	0.56^***^	0.07	0.42	0.67
Total indirect effects	0.22^***^	0.06	0.11	0.33
Direct effects	0.34^***^	0.08	0.19	0.50
*Specific indirect effects via*				
Amotivation	0.00	0.03	−0.06	0.06
Controlled motivation	−0.01	0.01	−0.06	0.00
Autonomous motivation	0.23^***^	0.05	0.14	0.33

95% BCa CI, bias corrected and accelerated confidence interval; β, standardized regression coefficient; *SD*, standard deviation; 2.50%, lower bound; 97.50%, upper bound.

*** *p* < 0.001 (two-tailed).

**Table 4:** The means (*M*), standard deviations (*SD*), and intercorrelations (*r*).

	*M*	*SD*	Purpose			Autonomous motivation			Work engagement		
			T1^a^	T2	T3	T1	T2	T3	T1	T2	T3
*Purpose*											
T1^a^	3.61	0.52	1								
T2	3.50	0.53	0.51^***^	1							
T3	3.51	0.59	0.49^***^	0.40^**^	1						
*Autonomous motivation*											
T1	3.76	0.59	0.37^**^	0.39^**^	0.05	1					
T2	3.69	0.74	0.14	0.27^**^	0.14	0.60^***^	1				
T3	3.54	0.71	0.23	0.17	0.25	0.30^*^	0.32^*^	1			
*Work engagement*											
T1	4.90	0.88	0.32^*^	0.28^*^	0.22	0.54^***^	0.30^*^	0.27^*^	1		
T2	4.79	1.04	0.09	0.23	0.16	0.47^***^	0.64^***^	0.28^*^	0.40^**^	1	
T3	4.60	0.93	0.93	0.25	0.43^**^	0.23	0.27^*^	0.66^***^	0.37^**^	0.45^***^	1

a *T1–T3* = Time-points 1, 2, 3; significance (two-tailed).

* *p* < 0.05;

** *p* < 0.01;

*** *p* < 0.001.

#### Structural Model

The variables purpose, autonomous motivation, and work engagement were specified in a cross-lagged panel model (CLPM) design, as depicted in Figure 2 and analyzed with Mplus 8, version 1.5(1). To estimate the model we followed Hamaker et al. (2015): the procedure was to specify the lagged and crossed effects, to make wave one endogenous, and to allow the residuals at the subsequent waves to be correlated, which resulted in the following model fit information: *χ* ^2^ (8) = 9.37, *p* = 0.31, *RMSEA* = 0.065, 90% CI [0.00 0.20], *CFI* = 0.985, *SRMR* = 0.047.

#### Hypothesis Testing

**Figure 4:**
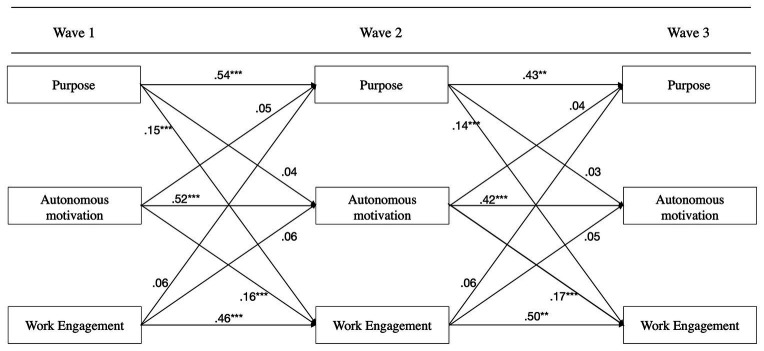
The crossed paths indicate directionality from purpose to engagement and from motivation to engagement. The crossed relationship between purpose and motivation is positive but nonsignificant.

Hypothesis 4 predicted a specific directionality from purpose to autonomous motivation and engagement and, hence, rule out the alternative direction from motivation or engagement to purpose. The stability of the means across time was checked through constraining the means for purpose at wave 1. A regular cross-lagged panel model returns the means at wave one and the intercepts for the subsequent waves, assuming the means to be constant over time by ignoring them and fitting the model to covariances only (McArdle and Nesselroade, 2014). The analysis indicated that the means were not constant, for which reason it was decided to estimate the model based on grand centered means (Hamaker et al., 2015). The data partly supported Hypothesis 4: the results (see Figure 4) indicated significant crossed relationships from purpose to engagement (wave 1–2: β = 0.15, *p* < 0.001; wave 2–3: β = 0.14, *p* < 0.001) and from motivation to engagement (wave 1–2: β = 0.16, *p* < 0.001; wave 2–3: β = 0.17, *p* < 0.001). For both crossed relationships, only the directions from purpose to engagement and motivation to engagement were significant. Between purpose and motivation, no significant crossed effects were found, although both directions were positive. These results signal a difference with the results of Study 1, where the association between purpose and autonomous motivation was significant.

## Discussion

Despite being frequently stated in popular media, the beneficial impact of a corporate purpose on employee motivation and engagement hardly receives attention in the current literature. In this study, we examined these relationships across two studies, using a cross-sectional and longitudinal design. In the first study, we found a positive cross-sectional association between purpose and autonomous motivation (Hypothesis 1a) and between purpose and engagement (Hypothesis 2). The structural model explained 56.7% of the variance in engagement with autonomous motivation as a mediator (Hypothesis 3a). Furthermore, purpose did associate negatively with amotivation (Hypothesis 1b), but, contrary to what we expected, positively, but nonsignificantly, with controlled motivation (Hypothesis 1c). Nor did amotivation or controlled motivation mediate the relationship between purpose and engagement (Hypotheses 3b and c).

The subsequent longitudinal study indicated a specific directionality in the relationship between purpose and engagement and between motivation and engagement. Purpose predicted subsequent employee engagement rather than the other way around (Hypothesis 4). The results highlight that employees who report being inspired by their organization’s higher purpose assert they are positively contributing to its realization. They also state they are striving to make the world a better place and are more engaged than others for whom the corporate purpose is less inspirational. The directionality supports the widespread assumption that a corporate purpose leads to engagement. Thus, a higher corporate purpose can be considered as an antecedent to work engagement, just as, for example, leadership (Carasco-Saul et al., 2015) and work climate (Bakker et al., 2007). Contrary to what we expected, and despite the positive and significant regression coefficients in the cross-sectional study, the longitudinal study did not corroborate the significant effect from purpose to autonomous motivation.

Organizations need engaged workers (Bakker and Schaufeli, 2008). Macey and Schneider (2008) maintain that work engagement constitutes a key to competitive advantage. Engagement also issues in the debate on the future of work, which is driven by rapid technological advancements such as artificial intelligence and big data (Arntz et al., 2020) and a growing interest in soft skills (Casillas et al., 2019). Still, work engagement remains a concern considering its current low levels (Gallup, 2017). The future of work debate occurs amidst growing political and economic complexities, fears over increasing inequalities, and unequal distribution of opportunities for learning and growth (cf. Jacobs and Mazzucato, 2016; ILO-Global Commission on the purpose of work, 2019). The formulation of a broader corporate purpose such as intimated in the press release of the Business Roundtable of August 2019 (BRT, 2019) and Fink’s letter to CEOs (Fink, 2019) might point to increasing awareness within corporations about their pivotal role in society (Husted, 2016). If this awareness translates into behaviors exemplifying the larger corporate purpose as described in the press announcement, then perhaps, after all, business can be a force for good (Polman, 2016).

The present study contributes to our knowledge of the impact of a broader corporate purpose on motivation and engagement. It has relevance considering the cultural beliefs about the centrality of work. A well-defined business objective taps into the deeply embedded cultural ideal of self-determination and authenticity (Taylor, 1991). Self-actualization through work has become an aspect of the modern sense of self-identity (Maslow, 1998). Contributing to serve a greater good supports eudaimonic well-being and nurtures the experience of authenticity (Ilies et al., 2005; Martela and Pessi, 2018).

The present study also adds to the leadership domain, as it expands our knowledge about the underlying process between a purpose as an aspect of good leadership and subsequent engagement (Inceoglu et al., 2018). Motivation, particularly autonomous motivation, explained most of the variance in work engagement in the cross-sectional study. Quite similar to the explanatory value of basic need satisfaction found in other studies into leadership and work engagement (e.g., Kovjanic et al., 2012; Rahmadani et al., 2019).

We did not find significant directionality in the relationship between purpose and motivation over time, but we maintain that studying the underlying process between leadership and engagement is very relevant. Within SDT, the interplay between motivation and purpose is part of the dialectical dynamic between a person’s sense of self and their social context. Perception-of-purpose and self-identity are constructs experienced at the personal level but by their very nature remain dynamic and are articulated in interaction with one’s cultural environment and social context. Taylor (1991, 2007) describes this dynamic between the individual’s sense of self and one’s cultural environment as dialogical or conversational. Other scholars have comparable ways of emphasizing the relational, conversational dynamic to explain the ontology of social and psychological phenomena (e.g., Winch, 1988; Maturana and Varela, 1992).

We suggest reaching beyond the corporate purpose itself, emphasizing the conversational essence of purpose and the importance of dialog between different stakeholders to make a purpose come to life (Bekke, 2006; Gartenberg et al., 2016). A corporate objective may play a role in mobilizing employees for a particular cause, and – as long as it aligns with the persons’ sense of self – it may bolster their self-identity and fuel autonomous motivation (Martela and Pessi, 2018). Hence, a corporate purpose may emerge as an essential element in attracting, selecting, and retaining employees who already have high levels of autonomous motivation and, consequently, find their autonomous motivation further enhanced through that specific purpose (Delaney and Royal, 2017).

The study also adds to leadership theory and responds to the current lack of research into corporate purpose. Finally, considering purpose as an aspect of good leadership (Shuck and Rose, 2013), the study contributes to the debate on the narrow preoccupation of organizational leadership with economic performance and its stress on increasing rationalization of business processes and efficiency at the expense of a normative, moral and ethical narrative (Freeman et al., 2018).

### Limitations and Suggestions for Future Research

The present study has some limitations. The results are based on a convenience sample from one multinational for-profit organization in a cross-sectional self-report survey. The subsequent longitudinal study had a small sample size. Another limitation is the absence of comparable studies on the effects of corporate purpose, so there was no reference material of other organizations to build on, limiting the potential for generalizations on the obtained study outcomes. To counter the limitations of the cross-sectional data, we followed the suggestions of Spector (2019). The subsequent CLPM analysis was conducted with grand centered means to correct the intermediate changes in the means (Hamaker et al., 2015).

A few suggestions for future research spring forward evaluating the present study and its limitations. First, it would be interesting to learn about the effects of comparable purposes of other organizations. Future research could also widen the scope and study the impact of different types of objectives, for instance, (1) a higher corporate purpose that stresses people, planet, and profit and (2) a shareholder value-oriented objective with centrality for profit maximization. Parmar et al. (2017) compared the impact of an inspiring stakeholder-oriented mission with a shareholder- and profit-oriented goal on the mean scores for autonomy, competence, and relatedness. The authors found significantly higher means for purpose over profit. We would expect such a typology to identify comparable associations between purpose, motivation, and engagement. Future studies could expand on the present outcomes and study the effects of a corporate purpose on other well-known variables such as turnover intentions, job satisfaction, and organizational citizenship behaviors.

The effects of a corporate purpose on meaning in life are of specific interest. Human beings yearn for meaning in their lives (Frankl, 2008; Wong, 2012). Work has become a place in life where people look for meaning and self-realization (Ciulla, 2000; Steger et al., 2012), but few people find true meaning through work (Mackey and Sisodia, 2014). As mentioned earlier in this paper, we found no studies into the specific effects of corporate purpose other than the work of Parmar et al. (2017). In contrast, there are many studies into meaningfulness and work as a source for meaning in life (e.g., Di Fabio and Blustein, 2016; Bailey et al., 2017; Lysova et al., 2019; Yeoman et al., 2019; Fremeaux and Pavageau, 2020). Some studies mention “broader” purpose (Martela and Pessi, 2018) as an antecedent to meaningfulness but do not specifically refer to corporate purpose.

Fourth, it would add to the knowledge on the interplay between purpose, motivation, and engagement to add qualitative studies based on interviews or focus groups as well as various longitudinal studies, preferably in an RCT setting. Presumably, engaging employees and leaders in a generative dialog around purpose and meaning will contribute to motivation and engagement. Lastly, we expect the associations of purpose, motivation, and engagement to differ between generational cohorts, considering the different work preferences and values younger generations bring to the workplace (Eversole et al., 2012; Lee and Edmondson, 2017).

### Practical Implications

From a more practical point, the study invites corporate leaders to rethink their organizations’ purpose beyond economic performance and revisit the significance of leadership for meaning-making through a broader objective, which used to be worthy of intellectual inquiry (Podolny et al., 2004). Even more so, because the underutilization of purpose as an instrument to sustainably motivate employees and drive work engagement (Keller, 2015) coincides with the crisis in work engagement (Mann and Harter, 2016) and sustainable motivation. Defining the organization’s objectives within a broader stakeholder perspective (Freeman et al., 2004) adds to employee engagement and brings organizational and performance benefits (Schneider et al., 2018). Additionally, a broader purpose may play a role in attracting and retaining talented workers (Delaney and Royal, 2017). It may appeal to younger generations, who are known to bring different work value preferences.

Below, we list some practical suggestions for a broader corporate objective mentioned in this study. It is not an exhaustive list but rather suggestions that foster autonomous motivation, engagement, and performance and which we consider worth reflecting. The first thing to evaluate is the firms’ broader corporate purpose beyond its financial strategy and performance. To what cause does the corporate purpose aim to contribute? Who (or what) does the firm consider as its stakeholders, and how are they involved? What does the firm do to propagate its purpose? Secondly, how are employees involved, and in what way does the firm’s purpose put people first? How does the purpose foster employee well-being and engagement, and what instruments are applied to stimulate that? Does the firm have processes and procedures to actively discuss its purpose with employees (and other stakeholders)? Additionally, a useful question to consider is whether and how employees feel inspired by the firm’s purpose and what may be needed to reinforce this? For example, by discussing to what extent employees feel they contribute to realizing the firm’s purpose through their work? Thirdly, how do communication around the purpose and its integration in current work processes support autonomous motivation? What is missing still? Moreover, does the purpose appeal to younger generations, considering that younger generations bring different preferences? Fourth, how does the firm specifically aim to benefit customers through its purpose? Fifth, how are the needs of society understood, and how does the firm’s purpose integrate those needs? Lastly, how does the purpose include and embrace ethics? How are ethics integrated into governance and current ways of working?

### Conclusion

The present study confirmed the widespread assumption that a higher corporate purpose leads to engagement. Whether or not autonomous motivation mediates this relationship is less clear. The cross-sectional study revealed a significant association between purpose and motivation, but the longitudinal study did not confirm its directionality. Autonomously motivated employees may find inspiration and further enhancement in an appealing, broader purpose; still, that purpose per se may not prove an antecedent to motivation. The paper answered the growing interest in the potential beneficial effects of a broader purpose, whereas the specific impact on motivation and engagement had not been studied before.

## Data Availability Statement

The datasets presented in this study can be found in online repositories. The names of the repository/repositories and accession number(s) can be found below: Data Archiving and Networked Services (DANS), on behalf of the Royal Netherlands Academy of Arts and Sciences (KNAW) Title: A corporate purpose as an antecedent to employee motivation and work engagement Persistent identifier: 10.17026/dans-zze-xsj2 Deposit date: 2020-05-05.

## Ethics Statement

The studies involving human participants were reviewed and approved by the board of the Faculty of Social and Behavioural Sciences, Utrecht University. Written informed consent for participation was not required for this study in accordance with the national legislation and the institutional requirements.

## Author Contributions

LT, WS, AB, and WR: conceptualization. LT: data analysis and data curation. LT and WS: writing – original draft preparation. LT, WS, AB, and WR: writing – review and editing. WS and WR: supervision. LT: project administration. All authors contributed to the article and approved the submitted version.

### Conflict of Interest

LT had a supplier agreement for coaching services at the organization where the data were gathered. He received no instructions, supervision or incentives from the organization pertaining to the research presented in this paper.

The remaining authors declare that the research was conducted in the absence of any commercial or financial relationships that could be construed as a potential conflict of interest.

## Footnotes

^1^For reasons of anonymity, the specific purpose of the organization is withheld as well as the name of the organization and any reference that may identify the organization.
